# *Anopheles gambiae* Antiviral Immune Response to Systemic O'nyong-nyong Infection

**DOI:** 10.1371/journal.pntd.0001565

**Published:** 2012-03-13

**Authors:** Joanna Waldock, Kenneth E. Olson, George K. Christophides

**Affiliations:** 1 Division of Cell and Molecular Biology, Department of Life Sciences, Imperial College, London, United Kingdom; 2 Arthropod Infectious Diseases Laboratory, Colorado State University, Fort Collins, Colorado, United States of America; Instituto Oswaldo Cruz, Fiocruz, Brazil

## Abstract

**Background:**

Mosquito-borne viral diseases cause significant burden in much of the developing world. Although host-virus interactions have been studied extensively in the vertebrate host, little is known about mosquito responses to viral infection. In contrast to mosquitoes of the Aedes and Culex genera, *Anopheles gambiae*, the principal vector of human malaria, naturally transmits very few arboviruses, the most important of which is O'nyong-nyong virus (ONNV). Here we have investigated the *A. gambiae* immune response to systemic ONNV infection using forward and reverse genetic approaches.

**Methodology/Principal Findings:**

We have used DNA microarrays to profile the transcriptional response of *A. gambiae* inoculated with ONNV and investigate the antiviral function of candidate genes through RNAi gene silencing assays. Our results demonstrate that *A. gambiae* responses to systemic viral infection involve genes covering all aspects of innate immunity including pathogen recognition, modulation of immune signalling, complement-mediated lysis/opsonisation and other immune effector mechanisms. Patterns of transcriptional regulation and co-infections of *A. gambiae* with ONNV and the rodent malaria parasite *Plasmodium berghei* suggest that hemolymph immune responses to viral infection are diverted away from melanisation. We show that four viral responsive genes encoding two putative recognition receptors, a galectin and an MD2-like receptor, and two effector lysozymes, function in limiting viral load.

**Conclusions/Significance:**

This study is the first step in elucidating the antiviral mechanisms of *A. gambiae* mosquitoes, and has revealed interesting differences between *A. gambiae* and other invertebrates. Our data suggest that mechanisms employed by *A. gambiae* are distinct from described invertebrate antiviral immunity to date, and involve the complement-like branch of the humoral immune response, supressing the melanisation response that is prominent in anti-parasitic immunity. The antiviral immune response in *A. gambiae* is thus composed of some key conserved mechanisms to target viral infection such as RNAi but includes other diverse and possibly species-specific mechanisms.

## Author Summary

Mosquito-borne viral diseases are found across the globe and are responsible for numerous severe human infections. In order to develop novel methods for prevention and treatment of these diseases, detailed understanding of the biology of viral infection and transmission is required. Little is known about invertebrate responses to infection in mosquito hosts. In this study we used a model system of *Anopheles gambiae* mosquitoes and *O'nyong-nyong* virus to study mosquito immune responses to infection. We examined the global transcriptional responses of *A. gambiae* to viral infection of the mosquito blood equivalent (the hemolymph) identifying a number of genes with immune functions that are switched on or off in response to infection, including complement-like proteins that circulate in the mosquito hemolymph. The switching on of these genes combined with co-infection experiments with malaria parasites suggests that viral infection inhibits the melanisation pathway. Through silencing the function of a selection of viral responsive genes, we identified four genes that have roles in *A. gambiae* anti-viral immunity; two putative recognition receptors (a galectin and an MD2-like receptor); two effector lysozymes. These molecules have previously non-described roles in antiviral immunity, and suggest uncharacterised mechanisms for targeting viral infection in *A. gambiae* mosquitoes.

## Introduction

Arthropod-borne viruses (arboviruses) are a significant health burden across the world. They represent an emerging and resurgent group of pathogens [1], many of which are transmitted by mosquitoes including Dengue Fever (DEN), Yellow Fever (YF), West Nile Virus (WNV) and Chikungunya (CHIKV). The development of control strategies to combat the spread of these viruses requires a detailed knowledge of host-pathogen interactions in both the vertebrate host and invertebrate vector. Targeting human pathogens, for example malaria parasites, within their insect vectors has been the focus of intense research towards identification of novel targets for transmission blocking interventions. Understanding the molecular mechanisms of immunity to pathogens within insect vectors could reveal potential candidates for such interventions.

Extensive research has been carried out into insect immune responses to bacterial, fungal and parasitic infections; however, it is only recently that invertebrate antiviral immunity has received analogous attention. Initial studies have used *Drosophila melanogaster* as a model system, as the power of genetics and the extensive knowledgebase in *Drosophila* have been invaluable in establishing the foundations for insect antiviral immunity research. However, the biology of arboviruses is tightly linked to the physiology of haematophagous arthropods, and as such research in model organisms may not be fully relevant to the transmission of viruses and associated vector defence. A forward research approach is required to effectively study the vector responses to arboviruses, utilizing findings in *Drosophila* as guidance.

Mosquitoes launch robust immune responses against a variety of pathogens: recognition of pathogen associated molecular patterns (PAMPS) leads to activation of immune signalling pathways associated with production of potent anti-microbial peptides (AMPs) or cascades that lead to pathogen lysis, phagocytosis, melanisation or cellular encapsulation by hemocytes, the white blood cell equivalents [2]. To date three signalling pathways have been implicated in mosquito antiviral immunity. The JAK/STAT pathway, a known antiviral signalling pathway in mammals [3], appears to have a conserved function in *Aedes aegypti*. JAK/STAT related genes are differentially regulated in response to DENV infection [4], and *Ae. aegypti* can be made more or less susceptible to DENV through silencing of *DOME* (receptor of the JAK/STAT pathway) and *PIAS* (negative regulator of the JAK/STAT pathway) respectively [5]. In addition, 18 genes downstream of the *Ae. aegypti* JAK/STAT pathway are regulated by DENV infection, two of which have been shown to be DENV antagonists [5]. The RNAi pathway has been demonstrated to limit viral infection in several mosquito vector-virus combinations. *AgAGO2* (a member of the RISC complex) is an antagonist of ONNV in *A. gambiae* [6]; *AeAGO2*, *AeDCR2* and *AeTSN* (all members of the RNAi pathway) are Sindbis virus (SINV) antagonists in *Ae. aegypti* [7]; *AeDCR2* had also been shown to be a DENV antagonist [8]. The presence of viRNA (siRNA that is specific to viral genomes) has been demonstrated in *Ae. aegypti* infected with SINV and DENV [9], [10], and recombinant viruses encoding suppressors of RNAi have been shown to increase mortality, increase viral titres and lower the build-up of viRNAs in infected mosquitoes [9], [10]. Finally, Toll pathway related genes are differentially regulated in response to both SINV and DENV infection in *Ae. aegypti* [4], [11]. Activation and inhibition of the Toll pathway has been demonstrated to respectively decrease and increase susceptibility to different DENV strains in different *Ae. aegypti* strains showing the importance of the Toll pathway in mosquito antiviral immunity [4], [12].

Whereas the *Aedes* and *Culex* mosquitoes transmit numerous viruses, *Anopheles* mosquitoes (the principal vectors of malaria) are known to be the primary vectors of only O'nyong-nyong virus (ONNV). ONNV is a positive (sense) strand single stranded RNA (+ssRNA) virus of the Alphavirus family, with reported epidemics in West Africa in the 1960s and 1990s [13]–[18]. Viral replication of ONNV in *A. gambiae* is shown to be slow and restricted in tissue tropism compared to most vector-virus combinations [19]. Permissiveness to infection has been shown, in part, to be regulated by RNAi, and inhibition of RNAi results in high susceptibility to viral infection [6]. Here we have profiled the global transcriptional responses of *A. gambiae* to ONNV infection of the hemolymph to identify viral responsive genes, and then used RNAi silencing to test a selection of identified genes for antiviral function. Our results confirm that in *A. gambiae* the RNAi pathway is a key antiviral mechanism, however, the JAK/STAT and the Toll pathway do not have a significant role in regulating systemic ONNV infection. We further identify four viral responsive genes with novel functions in mosquito antiviral immunity. Patterns of immune gene expression coupled with co-infections of *A. gambiae* with the rodent malaria parasite *P. berghei* suggest that viral infection inhibits parasite melanisation. Overall, we demonstrate that *A. gambiae* uses a combination of conserved antiviral pathways, including RNAi, and novel uncharacterised mechanisms to target ONNV infections.

## Materials and Methods

### ONNV production and propagation

5′ONNVic-eGFP plasmid was kindly provided by Dr Brian Foy, Colorado State University. 5′ONNCiv-eGFP infectious clones were generated as described in [19] with some modifications. RNA generated *in vitro* from the infectious clone template was purified using the RNeasy mini kit (Qiagen). RNA concentration and purity was ascertained using a Nanodrop (Labtech International). 2 µg of RNA were transfected into a confluent culture of VERO cells in a T75 flask using the Transmessenger transfection reagent (Qiagen). Cells were observed for cytopathic effects and GFP expression at 24 hours post transfection. At 72 hours post infection cells were scraped and filtered through a 0.22 µm filter, aliquoted and stored at −80°C. 250 µl of first passage 5′ONNVic-eGFP was used to infect a large culture of confluent VERO cells. At 72 hours post infection cells were scraped, filtered through a 0.22 µm filter, aliquoted and stored at −80°C. Second passage virus was used in all experiments.

### Maintainence of G3 *A. gambiae* mosquitoes

Adult mosquitoes were maintained as described in detail by Sinden and co-workers [20]. In brief, mosquitoes were reared and maintained at 28°C, 65–70% relative humidity with a 12 hour light/dark cycle. Adult mosquitoes were fed on sterile filtered and autoclaved 10% fructose solution and used for experimental purposes when 1 or 2 days old.

### Infection of adult G3 mosquitoes

Newly emerged female mosquitoes were inoculated with the required dilution of second passage 5′ONNVic-eGFP in MEM (Invitrogen), using a pulled capillary glass needle and a Nanoject (Drummond Scientific). Inoculated mosquitoes were kept in cohorts of 30–50 and maintained as described by [20]. Inoculated mosquitoes were double-contained to prevent escape.

### Qrt-PCR to assay viral titre in adult mosquitoes

Pools of ∼30 whole mosquitoes were homogenised in 200 µl of Drosophila Schneiders medium (Gibco). Homogenates were centrifuged at 3000 g for 30 minutes at 4°C to pellet debris. Supernatant was transferred to a new 1.5 ml eppendorf tube and centrifuged at 5000 rpm for a further 30 minutes at 4°C. The supernatant was filtered through a 0.2 um filter, and 140 µl of the filtrate was used for viral RNA extraction using the Qiagen viral RNA extraction kit according to the manufacturer's instructions. 10 µl of vRNA was used to generate cDNA using the Superscript II kit (Invitrogen). To ascertain the abundance of viral RNA, or viral genome copy number, an absolute quantification method was used. cDNA was generated from vRNA extracted from a sample with a known viral titre, calculated using standard plaque assay. A standard curve the sample was generated using neat, 1∶5, 1∶10. 1∶50, 1∶100 and 1∶500 dilutions of cDNA. Qrt-PCR was carried out using SybrGreen reagents (Applied Biosystems) and primers against the *nsP3* ONNV gene (Table S3). The standard curve was used to calculate the viral genome copy number of an unknown sample by mapping the CT value to that of the standard curve, giving the viral genome copy number.

### Qrt-PCR to assay gene KD efficiency

Total RNA was extracted from pools of ∼10 mosquitoes in TRIzol (Invitrogen) 4 days after dsRNA treatment. cDNA was generated from total RNA using the Superscript II kit (Invitrogen). Primers were designed against GOIs such that there was no overlap with dsRNA probes (Table S3) including the S7 gene that is constitutively expressed in the mosquito. Qrt-PCR was carried out using SybrGreen reagents (Applied Biosystems). cDNA input was normalised using the abundance of S7 in each sample. Once normalised, gene KD efficiency was calculated as a relative % decrease in transcript abundance compared to a control KD sample.

### Plaque assay

Standard plaque assays were carried out as described in [21]. In brief, individual mosquitoes were homogenised in 270 µl of Drosophila Schneiders medium (Gibco) and filtered through 0.22 µm filters. 10-fold serial dilutions of each sample were added in duplicate to confluent monolayers of VERO cells in 24 well plates and immobilised using an agar nutrient solution. Cells were stained after 4 days incubation at 37°C using 200 µl of 5 mg/ml Thiozolyl Blue Tetrazolium Bromide (MTT) (Sigma) in PBS. Plaques of dead cells were counted and used to calculate the plaque forming units (PFU)/mosquito.

### Microarray hybridisation and analysis

Total RNA extracted from whole homogenates of *A. gambiae* mosquitoes was amplified and labelled using the Low RNA Input Amplification kit (Agilent, UK). In brief: 2 µg of total RNA was used in a random primed reverse transcription reaction to generate cDNA. After amplification by conversion to cDNA, cDNA was transcribed to copy messenger RNA (cmRNA) incorporating either Cy-3UTP (for the reference sample) or Cy-5UTP (for the test sample) fluorescent nucleotide analogs. cmRNA quality and labelling efficiency was assessed by spectrophotometry using a Nanodrop (Labtech International). If cmRNA yield was sufficient and Cy-3UTP or Cy-5UTP labelling was successful, 825 ng of RNA was hybridised to the Agilent 4X44K array in 2× GEx-hybridisation buffer HI-RPM at 60°C for 17 hours. Hybridised slides were washed with GE wash buffer 1 at RT for one minute and GE wash buffer 2 at 37°C for one minute, to remove excess labelled cmRNA prior to scanning.

Microarrays were scanned using a GenePix semiconfocal microarray scanner (AXON Instruments, Foster City, CA) Gene Pix Pro 6.1 was used to record feature signal intensity, to eliminate local backgrounds, for grid alignment and manual inspection of feature quality. Average feature diameter was calculated and features lying outside three standard deviations of the mean were excluded from analysis. The ratio of feature intensity verses local and global backgrounds were calculated and features not exceeding background intensities were excluded from analysis. Features were normalised using Genespring 6.1 (Axon instruments) by locally weighted linear regression methods (Lowess). Feature intensities of the three biological replicates were averaged. T-test p-values were calculated, and normalised data was filtered to exclude data with p-values greater than 0.05. Data was further filtered to include only genes showing 2-fold and greater regulation. Candidate genes were selected based on several criteria, including gene ontology, and known roles of orthologous genes. Microarray data has been submitted to the open access Vectorbase database (www.vectorbase.org).

### Design and production of dsRNA probes

Primers were designed (Table S3) for 200–600 bp sections of genes of interest, with a T7 promotor sequence (GAATTAATACGACTCACTATAGGGAGA) added to their 5′ ends. Polymerase chain reaction (PCR) was carried out using cDNA derived from *A. gambiae* mosquitoes and PCR products were sequenced to confirm correct amplification for each probe. PCR amplicons were used to synthesise dsRNA using the T7 MEGAscript kit (Ambion). Concentration of dsRNA was adjusted to 3 µg/µl and stored at −80°C until use.

### Co-infections with *P.berghei*

*P. berghei* ANKA clone 259c12 was maintained in Theiler's original mice (Harlan, UK) as described in [20]. All animal work was carried out by Dr Tibebu Habetewold and Kasia Sala. Mice were infected by intraperitoneal (IP) injection of 100–200 µl of *P. berghei* infected blood. For mosquito infections, three days after passage with infected blood mice were terminally anaesthetised with an intramuscular (IM) injection of 0.05 ml/10 g body weight of Rompun (2% stock solution, Bayer), Ketastet (100 mg/ml ketamine, Fort Dodge Animal Health Ltd) and PBS in a 1∶2∶3 ratio.

Newly emerged adult G3 mosquitoes were intrathoracically inoculated with ∼1640 PFU 5′ONNVic-eGFP. Inoculated mosquitoes were maintained at 27°C for 48 h. Mosquitoes were starved of sugar for 4–5 hours prior to blood feeding. Mosquitoes were fed on a terminally anaesthetised *P. berghei* infected mouse, maintained at 19°C for 72 h post blood feeding to allow successful parasite development and were subsequent maintained at 27°C to allow for optimal viral replication. Unfed mosquitoes were removed between 24 and 48 h post blood feeding, when the blood bolus is clearly visible through the abdomen of the mosquito. Seven days post blood feeding, mosquito midguts were dissected and fixed in 4% PFA. Fixed midguts were mounted in Vectorshield (Vectorlabs) on glass slides with sealed coverslips. Live oocysts expressing GFP were counted using fluorescence, and melanised ookinetes were counted using light microscopy.

### Statistics

For plaque assay experiments and *P. berghei* oocysts/ookinete quantification, results were subject to the Man Whitney U-test for statistical significance. Significance was accepted where P<0.001***, P<0.01**, P<0.05*. For analysis of changes in *P. berghei* melanisation prevalence, results were subject to the Chi Squared test for statistical significance, where P<0.001***. Statistical significance in microarray experiments was calculated using the T-test comparing normalised (Lowess) expression data. Differential regulation was considered were fold change in expression was greater than 2 and P<0.05 over three biological replicates.

### Ethics statement and approval of experimental procedures

This study was carried out in strict accordance with the United Kingdom Animals (Scientific Procedures) Act 1986. The protocols for maintenance of mosquitoes by blood feeding and for infection of mosquitoes with *P. berghei* by blood feeding on parasite-infected mice were approved and carried out under the UK Home Office License PLL70/6347 awarded in January 2008 and PPL70/7185 awarded in November 2010. The procedures are of mild severity and the numbers of animals used are minimized by incorporation of the most economical protocols. Opportunities for reduction, refinement and replacement of animal experiments are constantly monitored and new protocols are implemented following approval by the Imperial College Ethical Review Committee. The experimental procedures for the ONNV work were approved by the HSE and the Imperial College GM Safety Committee.

## Results

### *A. gambiae* infections with ONNV

**Figure 1:**
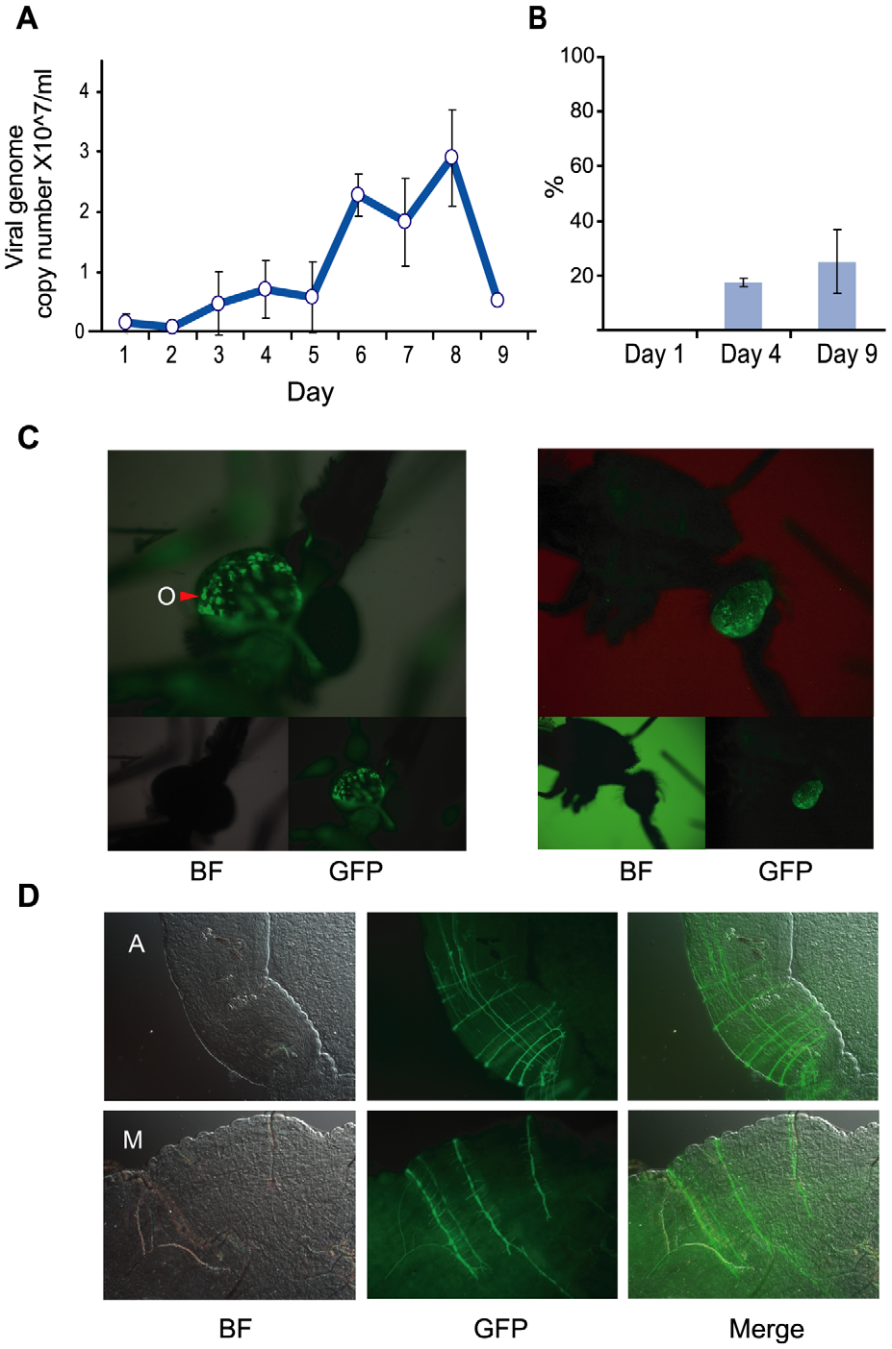
*A. gambiae* G3 mosquitoes intrathoracically inoculated with 5′ONNVic-eGFP. Mosquitoes were inoculated with ∼1650 PFU of 5′ONNVic-eGFP. (**A**) 10 inoculated mosquitoes were collected daily and qrt-PCR was used to ascertain viral genome copy number/mosquito. (**B**) Percent of inoculated mosquitoes showing GFP expression at 1, 4 and 9 DPI. (**C**) Brightfield (BF) and fluorescent (GFP) images of GFP expression in the head tissues through the ommatidia (O) of inoculated mosquitoes at 9 DPI and (**D**) Brightfield (BF) and fluorescent (GFP) images of GFP expression in nerves and/or muscle bands in the anterior- (A) and mid-gut (M) of inoculated mosquitoes. Error bars represent standard deviation of 3 biological replicates.

Using an infectious clone of ONNV encoding enhanced GFP (5′ONNVic-eGFP) under the control of a duplicated viral subgenomic promoter (provided by B. D. Foy, AIDL, Colorado state University [19]), we characterized infection of ONNV in adult G3 *A. gambiae* mosquitoes. Adult mosquitoes were intrathoracically inoculated with ∼1640 PFU/mosquito. Viral RNA (vRNA) was extracted from 10 pooled mosquitoes every day over 9 days and quantitative real-time PCR (qrt-PCR) was used to calculate the viral genome copy number/mosquito (Figure 1A). Viral titre increased slowly until 5 days post infection (DPI), when infection rapidly increased, peaking at 7–8DPI, and then subsequently decreased to low levels at 9DPI. Plaque assays using individual mosquitoes at 7DPI showed that the prevalence of infection was ∼90% (data not shown). GFP expression was also monitored at 1, 4 and 9 DPI by fluorescence microscopy of live, cold anaesthetized mosquitoes (Figure 1B). GFP expression, most commonly visible through the eyes (Figure 1C) and occasionally through the thorax, was visible in only ∼20% of mosquitoes at 4DPI and ∼25% of mosquitoes at 9DPI. The discrepancy in infection prevalence between plaque assays and GFP observations is attributed to the mosquito cuticle that provides a barrier to GFP detection and together with strong autofluorescence leads to underestimation of the infection prevalence in whole mosquitoes. In dissected mosquitoes at 7DPI, patterns of infection and tissue tropism were in agreement with those previously published using the same strain of *A. gambiae* mosquitoes and the same infectious clone of ONNV [19]. Additionally GFP expression was commonly seen in the midgut musculature of infected mosquitoes (Figure 1D) similar to what has been previously observed in other vector-alphavirus combinations [22], [23].

### Genome-wide transcriptional responses

**Figure 2:**
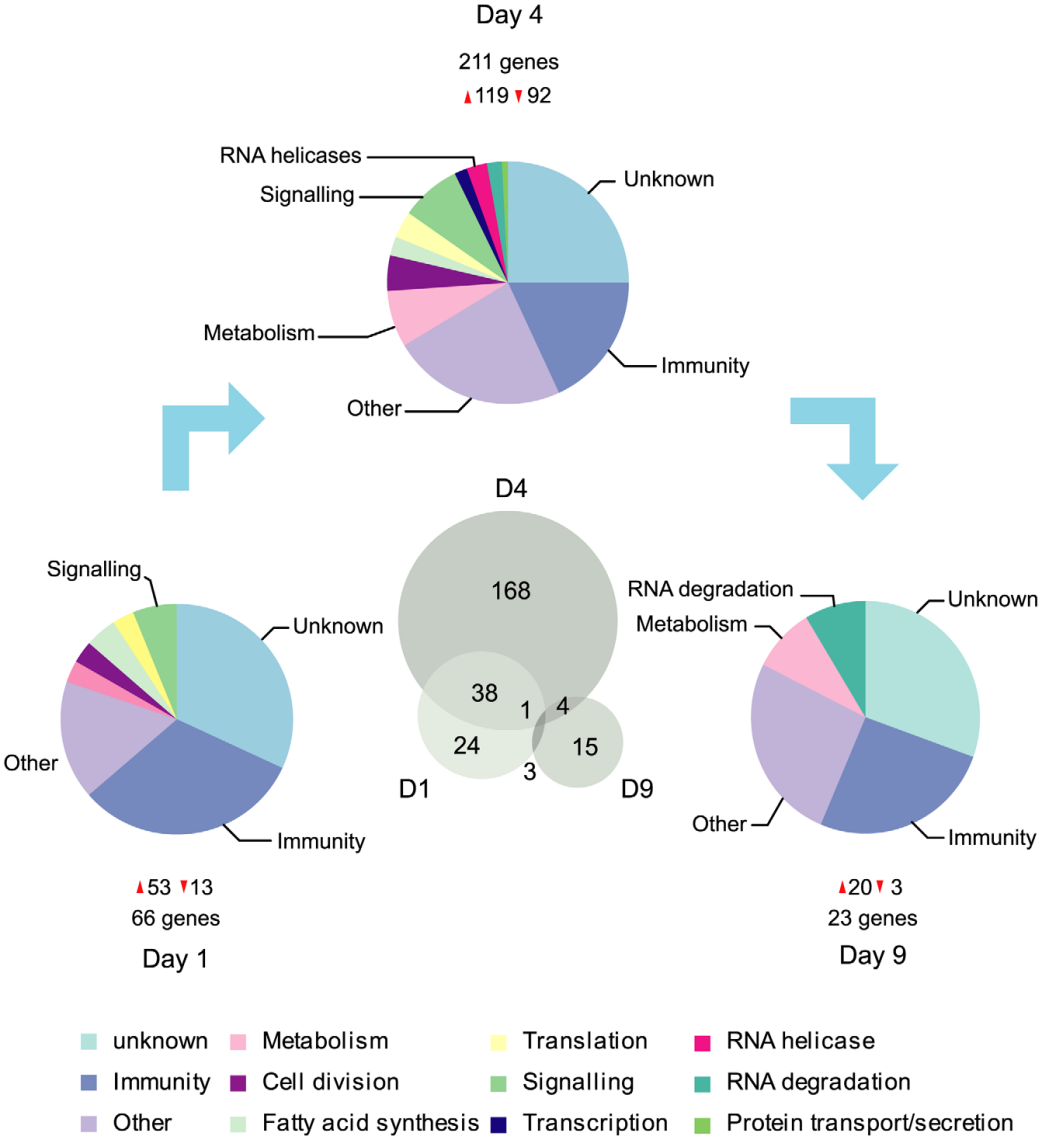
Transcriptional responses of *A. gambiae* G3 mosquitoes to 5′ONNVic-eGFP infection. The transcriptional responses of *A. gambiae* mosquitoes inoculated with ≈1640 PFU of 5′ONNVic-eGFP infection were profiled using 4X44K Agilent RNA microarrays. Gene lists include only features that pass strict criteria outlined in the materials and methods. Genes included the analysis are 2-fold or greater regulated at a minimum of 1 of the 3 time points, with T-test P values of <0.05. Genes were categorised based on gene ontology.

In order to investigate the responses of *A. gambiae* to systemic viral infection, we utilised a genome-wide microarray platform to profile gene expression during a time-course of ONNV infection of the hemocoel. Three time points were selected for analysis: 1DPI (representing initial introduction of virus into the hemocoel), 4DPI (where virus has replicated, is being released from infected cells and is infecting new tissues) and 9DPI (where infection levels have significantly dropped). Transcriptional profiling of whole mosquito homogenates from infected versus mock-infected mosquitoes revealed a large number of viral responsive genes. Initial exposure to virus (1DPI) triggered the differential regulation of 66 genes (53 upregulated and 13 downregulated), increasing to 211 genes (119 upregulated and 92 downregulated) at 4DPI and dropping to 23 genes (20 upregulated and 3 downregulated) at 9DPI (Figure 2). A full list of regulated genes is presented in Table S1. Genes were grouped into functional categories based on gene ontology (GO) terms, orthologous gene function and literature reviews. These categories span a wide range of cellular and physiological processes including metabolism, RNA degradation, signalling, and cell division; however, the most striking category pertains to genes with putative immune functions, particularly at 1DPI (30% of regulated genes at 1DPI, 18% at 4DPI and 26% at 9DPI).

Overall, 45 genes with putative immune functions were differentially regulated following ONNV infection. Grouping these genes based on gene ontology and putative function (Table S2) revealed genes with roles in all aspects of immunity, including pathogen recognition, complement-like proteins, immune signalling pathway components, humoral cascade regulators and effector genes. The majority of genes (39/45) were upregulated, consistent with the hypothesis that viral infection triggers immune signalling in *A. gambiae*.

A surprisingly small number of genes from immune signalling pathways known to respond to viral infection in other invertebrates were differentially regulated; comparison of viral responsive genes with those downstream of the Toll and IMD pathway in *A. gambiae* [24] and the JAK/STAT pathway in *A. gambiae* (unpublished data) demonstrated very little overlap in gene expression indicating that these pathways are not activated by ONNV infection. In fact at 4DPI genes involved in the RNAi, JAK/STAT and IMD pathways were downregulated suggesting inhibition of these signalling pathways. Downregulated genes encode the Tudor-SN (*TSN*), a component of the RNAi RISC complex, the janus kinase *HOP* of the JAK/STAT pathway, and *IKKb*, a positive regulator of the IMD pathway. In contrast to decreased *IKKb* transcripts, *DPT* and *CEC3* (AMPs thought to be downstream of the IMD pathway) were upregulated at 1DPI and 4DPI.

Genes encoding putative recognition receptors were upregulated at 1DPI and 4DPI; two MD2-like receptors (*ML1/9*), three galectins (*GALE6-8*), one fibrinogen-like protein (*FREP50*) and *GNBPB1* all increased in transcript abundance. Additionally a large number of genes encoding proteins implicated in humoral immunity were upregulated, consisting of LRIMs and complement-like Thioester-containing proteins (TEPs). Different LRIMs and TEPs were regulated during the different phases of infection; *LRIM1/4* and *TEP5* at 1DPI; *LRIM7* and *TEP4/9/10/12* at 1DPI and 4DPI; *LRIM10* and *TEP14* at 4DPI and *LRRD7* at 4DPI and 9DPI.

A number of clip-domain serine proteases and their inactive homologs (CLIPs) and C-type lectins (CTLs) were upregulated including two known inhibitors of melanisation (*CTLMA2* and *CLIPA2*). The roles of the other regulated CLIPs and CTLs are not known, although they probably function in the modulation of signalling that regulates humoral responses. Genes encoding additional putative immune effectors were upregulated, the majority of which at 4DPI, including two hydrogen peroxidases, a glutathione peroxidase and three lysozymes. Additionally apoptosis related genes also responded to viral infection. Downregulation of the inhibitor of apoptosis-1 (*IAP1*) and upregulation of Caspase-6 (*CASPS6*) at 4DPI suggests that apoptosis may be triggered.

### Gene silencing and ONNV infection phenotypic analysis

**Figure 3:**
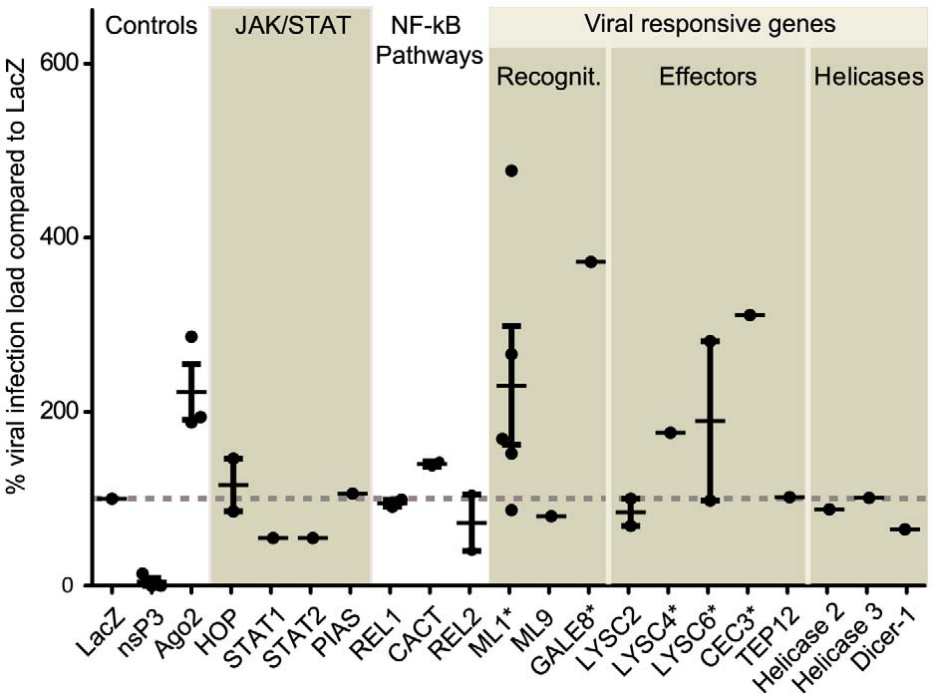
RNAi and qrt-PCR based screening of 19 genes for antiviral function in *A. gambiae* mosquitoes. Mosquitoes were injected with dsRNA and ∼3000 PFU of 5′ONNVie-eGFP concurrently, 30 mosquitoes were collected at 7 DPI, and qrt-PCR was used to ascertain viral genome copy number/mosquito. Values were normalised relative to the *LacZ* (non-specific) control and are given as a percentage relative to the LacZ titre. *nsP3*, a viral gene was included in the screen. Genes are divided into functional categories based on which immune signalling pathway they belong to, or their putative function. Genes with an asterisk* were selected for further characterisation.

Transcriptional profiling highlighted immune genes that respond to infection, however, whether these genes have genuine antiviral functions could not be inferred from expression profiling alone. To identify genes that have roles in *A. gambiae* antiviral immunity we developed an RNAi and qrt-PCR based assay to measure the effects of gene knockdown (KD) on viral titres. Mosquitoes were co-inoculated with dsRNA corresponding to a gene of interest and ∼3000 PFU of 5′ONNVic-eGFP. The viral RNA genome copy number per mosquito was calculated 7DPI using qrt-PCR. 19 genes were selected from the viral responsive immune genes identified in our transcriptional analysis and from the classical immune signalling pathways. DsRNA corresponding to *AgAGO2* and the ONNV *nsP3* gene were included as positive and negative controls respectively, while dsRNA corresponding to the *Escherichia coli LacZ* gene was used as a reference to calculate percentage changes in viral infection loads. As expected KD of *AgAGO2* resulted in increased 5′ONNVic-eGFP titres and *nsP3* silencing resulted in decreased 5′ONNVic-eGFP titres, indicating that our screening method was accurate (Figure 3). Interestingly, silencing of genes from the JAK/STAT and the Toll pathways (*HOP*, *STAT1-2*, *PIAS*, *REL1*, *CACT*), which are known to be involved in antiviral immunity in *Ae. aegypti* and *D. melanogaster*, as well as of the IMD pathway (*REL2*) did not have an effect on 5′ONNVic-eGFP titres or yielded data that were highly variable and therefore inconclusive. However, 5 of the 10 tested viral responsive immune genes identified in our gene profiling experiments appeared to be viral antagonists and were selected for further investigation: the putative recognition receptors *ML1* and *GALE8*, and the antimicrobial peptides *LYSC4*, *LYSC6* and *CEC3*.

**Figure 4:**
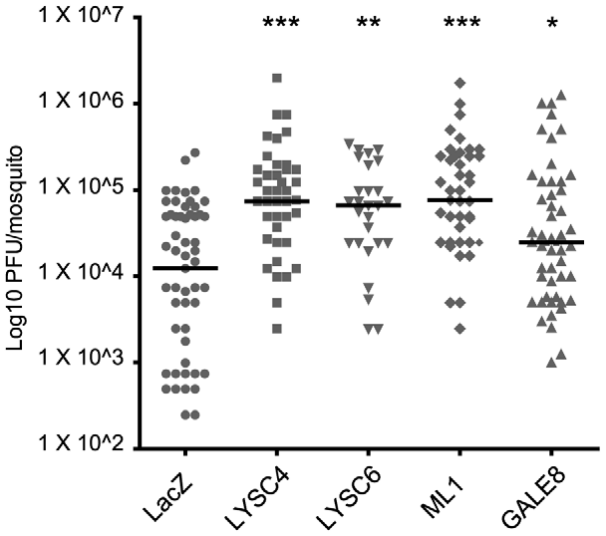
Four antagonists of 5′ONNVic-eGFP in *A. gambiae* mosquitoes. Mosquitoes were injected with dsRNA and ∼3000 PFU/mosquito P1/P2V 5′ONNVic-eGFP concurrently. Individual mosquitoes were collected and subject the plaque assay at 7 DPI. Data was Log10 transformed. Bars represent the median from two independent biological replicates. P values indicate significance from Man Whitney U Testing of each gene KD compared to the LacZ control (P<0.05*, P<0.01**, P<0.001***).

In order to confirm that the 5 genes identified in our qrt-PCR screen have a statistically significant impact on ONNV infection we carried out plaque assays to measure the viral titres in individual gene silenced mosquitoes. Silencing 4 of the 5 tested genes consistently affected viral titres (Figure 4). *ML1* KD resulted in a 6.2 fold increase (P<0.0001), *LYSC4* KD resulted in a 6 fold increase (P<0.0001), *LYSC6* KD resulted in a 5.4 fold increase (P<0.001) and *GALE8* KD resulted in a 2 fold increase (P = 0.0163) in median viral titres at 7DPI. The prevalence of infection was similar for all genes tested (*LacZ* 89%, *ML1* KD 95%, *GALE8* KD 95%, *LYSC4* KD 89% and *LYSC6* KD 93%). Qrt-PCR was used to confirm the reduction of *ML1* (91%), *GALE8* (80%) and *LYSC4* (82%) transcripts in mosquitoes after dsRNA treatment and to confirm the transcriptional profile of these genes after systemic infection (Figure S1); *LYSC6* was not assayed.

### *A. gambiae* co-infection with ONNV and *P. berghei*

Several recent studies have shown that the *A. gambiae* humoral immune system exists in a delicate state of balance that can be diverted to lysis or melanisation against malaria parasites [25], [26]. To investigate whether the differential regulation of several humoral immune factors observed during infection with ONNV, including CLIPs, CTLs, LRIMs and TEPs, can influence this balance we utilised *A. gambiae* co-infections with ONNV and the rodent malaria, *P. berghei*.

Newly emerged female mosquitoes were injected with ∼1640 PFU 5′ONNVic-eGFP or mock infected and blood-fed 48 h later on a mouse infected with *P. berghei*. This experimental design resulted in parasite ookinetes traversing the midgut wall and entering the hemolymph approximately 4 days post ONNV infection. Seven days post blood-feeding the mosquito midguts were dissected and parasite oocysts and melanised ookinetes were counted. Additionally, midguts were scored for ONNV infection of the midgut musculature.

**Figure 5:**
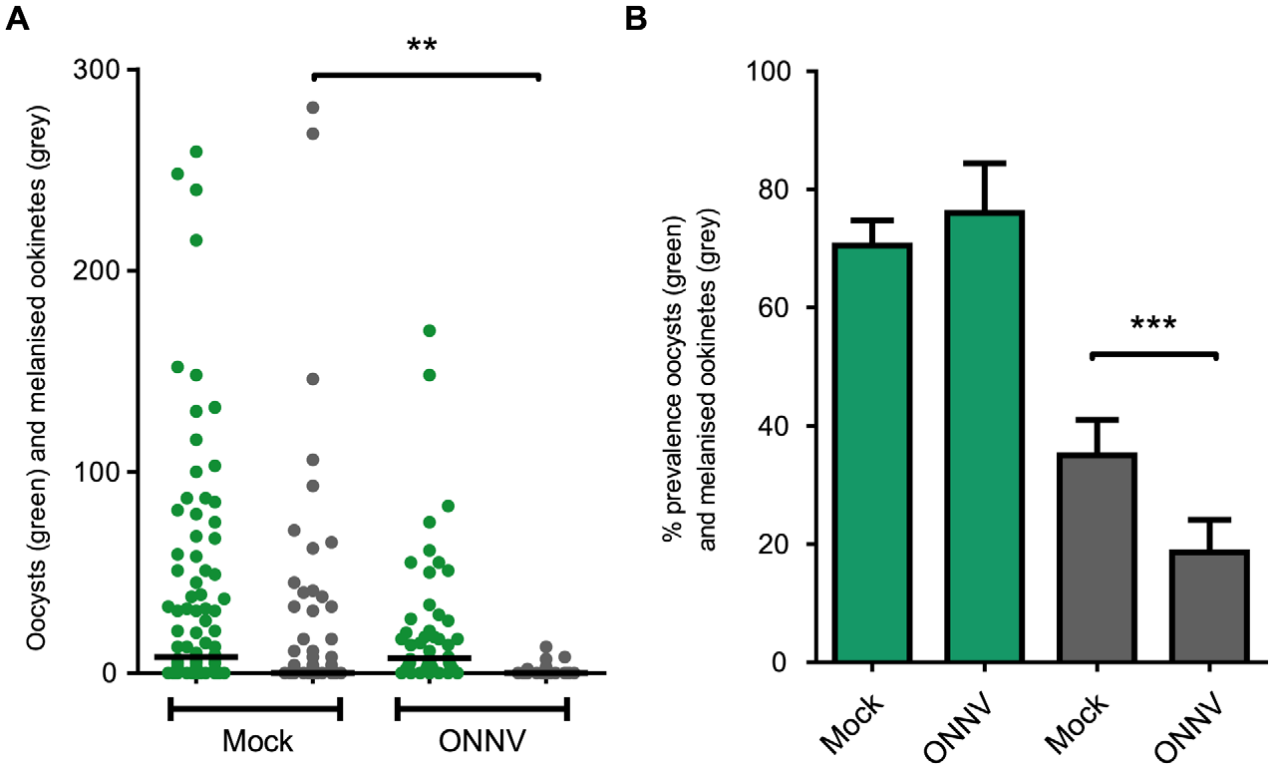
Co-infections of *A. gambiae* with *P.berghei* and 5′ONNVic-eGFP. G3 mosquitoes were inoculated with ∼1640 PFU 5′ONNVic-eGFP (+) or mock inoculated (−). 48 hours later mosquitoes were fed on a mouse infected with *P. berghei*. (**A**) 7 days post blood feeding midguts were dissected, live oocysts (green)/melanised ookinetes (grey) were counted, and guts were scored positive or negative for 5′ONNVic-eGFP expression in midgut musculature or nerves. Parasite numbers were only included for the+virus category when 5′ONNVic-eGFP expression was observed. Median values for three biological replicates are represented by a black bar. Statistical significance was assessed using the Mann Whitney U test of three biological replicates (**P<0.01). (**B**) Prevalence of parasite infection at 7 dpi in uninfected (mock) and infected (ONNV) midguts. Error bars represent standard error of three biological replicates. Statistical significance was assessed using the Chi square test (***P<0.001).

Figure 5A shows the oocyst and melanised ookinete distribution in virally infected and mock infected mosquitoes. The results revealed that priming with ONNV results in an approximately 40% reduction in the number of live oocysts in the virally infected mosquitoes showing viral midgut infections, although this decrease was not statistically significant using the Man Whitney test. However, a statistically significant decrease in the numbers of melanised ookinetes was observed (P = 0.001). The prevalence of melanised ookinetes also significantly decreased from 35% in mock infected to 18.5% in virally infected mosquitoes (Figure 5B) (P<0.001 using the Chi squared test).

## Discussion

Viruses as obligate intracellular pathogens represent a unique challenge to the immune system and require sophisticated mechanisms of recognition and targeting. Completing their lifecycle within host cells and use of host cell membranes limits the number of signatures that the immune system can recognise as non-self. In this study we have attempted to elucidate the components of the immune system employed by *A. gambiae* mosquitoes to target ONNV through transcriptional profiling of infected mosquitoes and gene silencing experiments.

Our observations of slow viral replication and restricted tissue tropism within the *A. gambiae* mosquito host are consistent with those observed for ONNV infections in the past [19]; it has been suggested that the robust RNAi response observed in *A. gambiae* mosquitoes [27] may contribute to the poor vectorial capacity of these mosquitoes in comparison with other typical vector-virus combinations [6]. Although not carried out in this study, dissemination rates for ONNV infection in *A. gambiae* have also been shown to be low compared to other vector-virus combinations [19]. This indicates that *A. gambiae* is a poor vector of ONNV, and may not be the natural vector of the disease outside of epidemics [28].

Our transcriptional profiling of ONNV infected mosquitoes has identified a large number of viral responsive genes. A significant proportion of these genes have no known function, indicating that *A. gambiae* may utilise non-classical immune mechanisms to target viral infection. Of the putative immune genes that were viral responsive, a perhaps surprisingly small number were associated with the Toll or JAK/STAT pathways. It has been shown in *D. melanogaster* and *Ae. aegypti* that both the Toll and JAK/STAT pathways have important roles in antiviral immunity [4], [5], [29]. In our study ONNV infection fails to induce expression of components of these two pathways; in fact *HOP* is downregulated at 4DPI. Additionally, there is very little overlap between viral responsive genes and those known to be downstream of the Toll pathway in *A. gambiae* [24] and genes identified through microarray analysis of *HOP* KD *A. gambiae* mosquitoes (unpublished data). Indeed through our gene silencing experiments we have observed that activating or inhibiting both pathways has little effect on ONNV titres. These data indicate that not only does systemic ONNV infection fail to trigger Toll and JAK/STAT signalling, but that genes downstream of these two pathways do not target ONNV infection.

A possible contributing factor to the surprising difference observed between *Ae. aegypti* and *A. gambiae* responses to viral infection may be the route of infection used in the experimental designs. The JAK/STAT and the Toll pathways have been shown to be important in regulating flavivirus DENV midgut infection after an infectious blood meal, which is the natural route of infection. In our study, intrathoracic inoculation was used to infect mosquitoes with ONNV in order to overcome the restricted tissue tropism and very limited dissemination rates observed following oral infection with ONNV, thus achieving higher levels and prevalence of systemic infection. Although a role of the JAK/STAT and the Toll pathway in midgut defense against ONNV is possible, our results demonstrate that once the virus enters the mosquito homocoel, immune responses other than the JAK/STAT and the Toll pathways are involved in regulating systemic antiviral immunity. Other studies investigating immune responses of insects to alphavirus infection have also suggested or demonstrated that the JAK/STAT and Toll signalling pathways do not target alphaviral infection. Sanders et al [11] conducted microarray analysis of SINV infected *Ae. aegypti*; despite suggesting a role for the Toll pathway during early infection, they found no differential regulation of JAK/STAT or Toll pathway components. Fragkoudis *et al* (2008) [30] found that SFV in all likelihood does not trigger classical immune signalling pathways in *Ae. albopictus* cells, and in fact infection inhibits activation of the JAK/STAT, Toll and IMD pathways, probably through reducing host cell gene expression. The difference observed between immune gene regulation and function using *Ae. aegypti*/DENV and *A. gambiae*/ONNV may also be a feature of flavivirus verses alphavirus infection respectively.

In addition to Toll signalling, a second NF-κB signalling pathway, the IMD pathway, regulates numerous genes that target *Plasmodium* parasites and bacteria [24], [31]. Our expression profiling shows that *IKKb*, a positive regulator of the IMD pathway, is downregulated at 4DPI, suggesting that the pathway may be inhibited. In contrast, upregulation of *LRIM1* and *CEC3*, which are downstream targets of the *A. gambiae* IMD pathway [31], as well as of a homolog of the AMP *Diptericin* that is downstream of the IMD pathway in *Drosophila* [32], may suggest activation of the pathway. Indeed a previous study has demonstrated that the IMD, but not the Toll pathway, has an antiviral function during SIN infection of Drosophila [33]. It is possible that the IMD, and not the Toll pathway, responds to alphavirus infection, with the reverse being true for flavivirus infection. Nevertheless, silencing *REL2*, the NF-κB factor of the IMD pathway, has no significant effect on ONNV titres, suggesting that the contribution of this pathway to ONNV infection, whether inhibited or activated, is minor.

RNAi has been demonstrated in a number of invertebrates to target and limit viral infection [6]–[10]. Our transcriptional profiling does not show any induction of expression of RNAi components, similar to observations of *D. melanogaster* infected with DCV [29] and *Ae. aegypti* infected with DENV [4]. Presumably the components of the RNAi pathway are constitutively expressed to levels sufficient to target replicating viruses. We confirm that RNAi is a key antiviral mechanism in *A. gambiae* mosquitoes through silencing of the *nsP3* viral gene and inhibition of RNAi by silencing *AgAGO2*. Interestingly transcriptional profiling revealed that *TSN*, a component of the RISC complex in the classical RNAi pathway, is downregulated at 4DPI. It is possible that this downregulation is mediated by ONNV, as inhibition of RNAi would be advantageous for infection. ONNV and other alphaviruses are thought not to encode a direct suppressor of RNAi [34] as seen in other insect viruses such as the *B2* protein of the FHV (Flock House Virus) [35]; however, it is possible that viral gene products may interfere with host gene expression. For example, 90% of Semliki Forest Virus (SFV; a closely related alphavirus) *nsP2* protein localises to the nucleus of infected cells where its function is unknown but could modulate expression of host genes [36].

In addition to the downregulation of *TSN*, two further genes involved in the miRNA and piRNA pathways, *DCR1* and *AgAGO5*, and a group of DEAD-box helicases are downregulated at 4DPI. There is evidence that the piRNA pathway has antiviral functions in *Drosophila*, as *Piwi* mutant flies are more susceptible to WNV than wild-type flies [37]. The DEAD-box helicases, although having diverse functions in RNA metabolism, are closely related to *RIG-I* and the RIG-I-like receptors (RLRs) that have well defined roles in viral RNA recognition in mammalian systems [38]. In addition, the human DEAD-box helicase *DDX3X* also has antiviral roles, and multiple viruses have been shown to interact with this protein and modulate its function [39]. The downregulation of these genes suggests an intriguing function in antiviral immunity. However, silencing of three helicases, including *DCR1*, has no effect on ONNV titre.

In addition to the immune signalling pathways, *A. gambiae* mosquitoes have a humoral branch of the immune system that recognises and eliminates invading pathogens. A ternary complex of two proteins of the LRIM family, *LRIM1* and *APL1C*, and the complement-like *TEP1* has been shown to target invading *Plasmodium* parasites for lysis or melanisation [25], [40], [41]. As our transcriptional analysis showed that *LRIM1* as well as several other members of the LRIM and TEP families are upregulated following infection, we investigated whether these humoral responses triggered by viral infection can interfere with *Plasmodium* infections. ONNV and *P. berghei* co-infections of *A. gambiae* were timed such that parasites traverse the midgut and enter the hemolymph 4 days after ONNV infection, thus encountering virus-induced humoral immune responses in the hemolymph. Our results reveal that parasite melanisation is significantly inhibited in the presence of ONNV. These results are consistent with our transcriptional analysis that shows upregulation of two important negative regulators of melanisation at 4DPI: *CTLMA2* and *CLIPA2* [42], [43]. An observed simultaneous decrease in the number of surviving parasites is not dramatic suggesting that upregulation of *LRIM1* alone is not sufficient to cause significant parasite lysis, and that parallel upregulation of *APL1C* and the complement effector protein *TEP1* would be needed. However, among additional TEPs upregulated by ONNV infection are *TEP4* and *TEP9*, which have been recently shown to also form complexes with *LRIM1* [44]. It remains to be investigated whether the alternative *LRIM1*/TEP complexes promote antiviral responses such as virus lysis or clearance of infected cells.

Four novel ONNV antagonists have been identified through our RNAi screen; *ML1*, *GALE8*, *LYSC4* and *LYSC6*. *ML1* is one of two MD-2 like receptors upregulated by ONNV infection and a known *P. falciparum* antagonist [45]. The *MD2* protein forms part of an LPS sensing mechanism in mammals [46], [47]. In addition to responses to LPS, *MD2*-*TLR4* signalling triggers the expression of pro-inflammatory cytokines in response to ebola envelope protein [48]. *TLR4* signalling has also been linked to several other viral infections including the vesicular stomatitus virus, respiratory syncytial virus, mouse mammary tumor virus [48] and Kaposi sarcoma herpesvirus [49]; however the role of *MD2* in these interactions is not clear. A TLR binding partner for the MLs has yet to be identified in flies and mosquitoes. One hypothesis is that the ML proteins may act as an extracellular surveillance system that recognise viral PAMPs and lead to signalling via a Toll receptor. At least 10 Toll receptors have been identified to date in *A. gambiae*, but their role in immune responses is yet unclear [50].

Beta-galactoside binding galectins are found in many organisms and display a complex repertoire: the multiple isoforms and their observed plasticity in sugar binding suggests substantial diversity in their glycan recognition properties [51]. There are 10 putative galectins in *A. gambiae* [50], 3 of which are upregulated by ONNV infection, *GALE6*-*8*. All three galectins are part of a mosquito specific expansion of the Galectin family, including also *GALE4* and *GALE5*. This expansion maybe due to the haematophagous lifestyle of mosquitoes, and subsequent exposure to a disparate group of blood-borne pathogens compared to *D. melanogaster*, including viruses. Therefore, the upregulation of several galectins in response to ONNV infection suggests that this group of mosquito specific galectins have antiviral roles. Galectins are known to function at several levels of antiviral defence, from initial recognition and blocking of envelope and fusion glycoproteins to the activation and amplification of the innate and adaptive immune responses [51]. In mammals, *Galectin 1* cross-links the N-glycans displayed in the envelope proteins of Nipah and Hendra viruses (paramyxoviruses that induce syncytia in infected cells), directly blocking cell infection and cell-cell fusion [51]. In addition, Galectin expression is regulated by Herpesvirus 1, Newcastle disease [52], Epstein Barr Virus [53], Hepatitus C virus [54] and Human papiloma virus (HPV) [55] while *Galectin 3* secretion and carbohydrate binding increase upon Herpesvirus 1 infection [56].

Three lysozymes are also induced by ONNV infection and two of those, *LYSC4* and *LYSC6*, appear to have antagonistic effects against ONNV infections. Although lysozymes are classical antibacterial proteins that function through perturbation of cell membranes [57], many of them are also shown to have antiviral immune functions including human urinary Lysozyme C, a lysozyme from chicken egg whites, human milk lysozyme and human neutrophil lysozyme, all of which have anti-Human Immunodeficiency Virus (HIV) activity [58]. In addition, a lysozyme from a marine organism is shown to inhibit Pseudo Rabies Virus (PRV) growth in cell culture [59]. Although, the mechanism of lysozyme antiviral activity is not clear, it is likely that they also function through membrane perturbation.

In summary, this study is the first step in elucidating the antiviral mechanisms of *A. gambiae* mosquitoes, and has revealed interesting differences between *A. gambiae* and other invertebrates. The finding that two pathways with known antiviral roles in other invertebrate-virus systems do not significantly modulate systemic ONNV infection indicates that *A. gambiae* may use other immune mechanisms to recognise and fight viral infections. Our data suggest that these mechanisms involve the complement-like branch of the humoral immune response, and that the melanisation response that is prominent in anti-parasitic immunity is suppressed. The antiviral immune response in *A. gambiae* is thus composed of some key conserved mechanisms to target viral infection such as RNAi but includes other diverse and possibly species-specific mechanisms.

## Supporting Information

Figure S1 **Qrt-PCR confirmation of virally responsive genes.** The expression of 3 virally responsive genes ascertained by microarray analysis (white diamonds) was confirmed using qrt-PCR (black diamonds). cDNA was generated from RNA extracted from 5′ONNVic-eGFP infected and mock infected (LacZ control) mosquitoes. Transcript levels are expressed as the fold change of those observed in the LacZ control. Error bars represent standard deviation of 3 biological replicates.
(TIF)

Table S1 **Genes differentially regulated by 5′ONNVic-eGFP infection.** Two-fold or greater fold change ratios are shown in black text for 1DPI, 4DPI and 9DPI. Fold change ratios less than 2-fold regulated that have passed all filters outlines in the materials and methods excluding filtering on fold change ratio, are shown in grey text. Putative functions/functional domains were derived from Gene ontology terms, Interpro domains and functions of orthologous genes (www.vectorbase.org).
(DOC)

Table S2 **Immune genes regulated by viral infection at days 1,4 and 9 post infection.** Two-fold or greater fold change ratios are shown in black text for 1DPI, 4DPI and 9DPI).. Fold change ratios less than 2-fold regulated that have passed all filters outlines in the materials and methods excluding filtering on fold change ratio, are shown in grey text. Putative functions/functional domains were derived from Gene ontology terms, Interpro domains and functions of orthologous genes (www.vectorbase.org).
(DOCX)

Table S3 **Primers used for A. qrt-PCR and B. RNAi probe generation.** **A.** Primers were designed using Primer3 for 50–150 bp sections of genes of interest. **B.** Primers were designed for 200–600 bp sections of genes of interest, with a T7 promotor sequence (GAATTAATACGACTCACTATAGGGAGA) added to their 5′ ends, ensuring no overlap between sections used for designing qrt-PCR primers.
(DOC)
