# Supplementary figures and images for: Anopheles gambiae Antiviral Immune Response to Systemic O'nyong-nyong Infection
Source: PLoS Negl Trop Dis. 2012 Mar 13;6(3):e1565. doi: 10.1371/journal.pntd.0001565 (PMC3302841; doi:10.1371/journal.pntd.0001565)

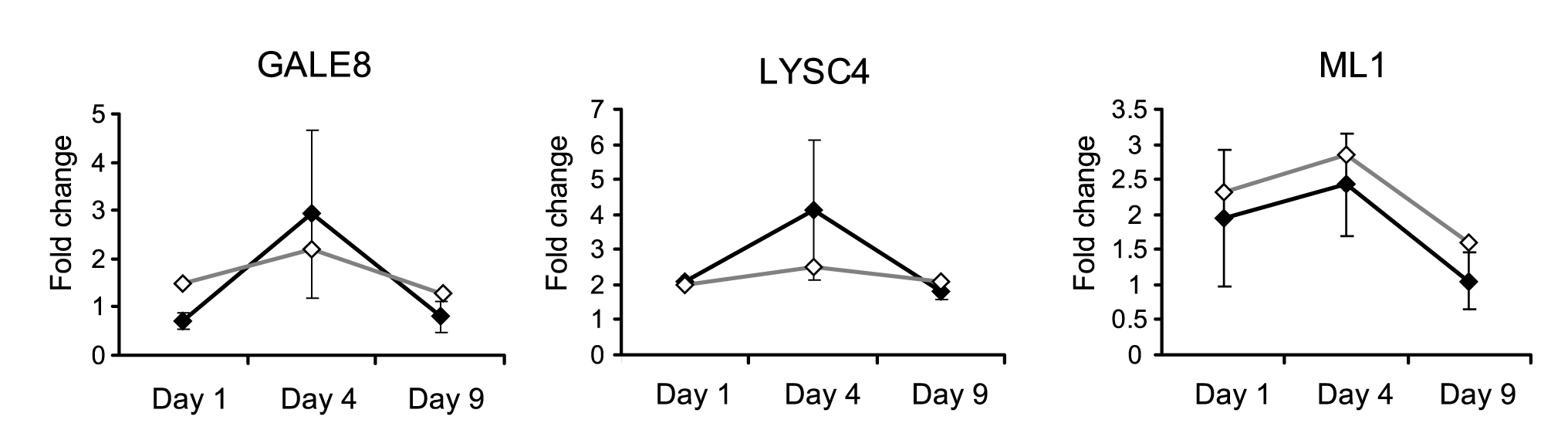

Supplement: Figure S1 — Qrt-PCR confirmation of virally responsive genes. The expression of 3 virally responsive genes ascertained by microarray analysis (white diamonds) was confirmed using qrt-PCR (black diamonds). cDNA was generated from RNA extracted from 5′ONNVic-eGFP infected and mock infected (LacZ control) mosquitoes. Transcript levels are expressed as the fold change of those observed in the LacZ control. Error bars represent standard deviation of 3 biological replicates. (TIF) [file pntd.0001565.s001.tif]
