# Supplementary material for: Anopheles gambiae Antiviral Immune Response to Systemic O'nyong-nyong Infection
Source: PLoS Negl Trop Dis. 2012 Mar 13;6(3):e1565. doi: 10.1371/journal.pntd.0001565 (PMC3302841; doi:10.1371/journal.pntd.0001565)
Supplement: Table S1 — Genes differentially regulated by 5′ONNVic-eGFP infection. Two-fold or greater fold change ratios are shown in black text for 1DPI, 4DPI and 9DPI. Fold change ratios less than 2-fold regulated that have passed all filters outlines in the materials and methods excluding filtering on fold change ratio, are shown in grey text. Putative functions/functional domains were derived from Gene ontology terms, Interpro domains and functions of orthologous genes (www.vectorbase.org). (DOC) [file pntd.0001565.s002.doc]

**Table S1. Genes differentially regulated by 5’ONNVic-eGFP infection**.

| AGAP | D1 | D4 | D9 | Common name | Functional group | Putative function |
| --- | --- | --- | --- | --- | --- | --- |
| **1DPI** |  |  |  |  |  |  |
| AGAP001899 | 2.03 |  |  |  | Fatty acid synthesis | Fatty acid synthase, putative |
| AGAP000260 | 2.08 |  |  |  | Housekeeping | ATP synthase epsilon subunit, putative |
| AGAP010814 | 2.60 |  |  | TEP5 | Immunity | Thioester containing protein |
| AGAP006348 | 2.28 |  |  | LRIM1 | immunity | Leucine rich repeat immune protein |
| AGAP004248 | 2.27 |  |  | GPXH3 | Immunity | Glutathione peroxidise |
| AGAP002848 | 2.01 |  |  | ML9 | Immunity | MD2-like receptor |
| AGAP007039 | 2.68 |  |  | LRIM4 | Immunity | Leucine rich repeat immune protein |
| AGAP005901 | 0.48 |  |  |  | Immunity | Sarm-1, putative |
| AGAP001116 | 2.02 |  |  |  | metabolism | FAD dependent oxidoreductase, putative |
| AGAP006009 | 2.49 |  |  | CPR30 | Misc | Insect cuticle protein |
| AGAP006958 | 0.48 |  |  |  | Misc | Heat shock protein, putative |
| AGAP010895 | 0.46 | 0.51 |  |  | Misc | Spectrin beta chain, putative |
| AGAP005913 | 0.45 |  |  |  | Signalling | WD-40 repeat containing G-protein, putative |
| AGAP005912 | 0.45 |  |  |  | Signalling | WD-40 repeat containing G-protein, putative |
| AGAP005911 | 0.39 |  |  |  | Signalling | WD-40 repeat containing G-protein,putative |
| AGAP002171 | 0.48 |  |  |  | Translation | Nucleolar protein |
| AGAP003773 | 4.37 |  |  |  | unknown | Conserved hypothetical protein |
| AGAP003778 | 4.12 |  |  |  | unknown | Conserved hypothetical protein |
| AGAP003777 | 4.06 |  |  |  | unknown | Conserved hypothetical protein |
| AGAP003939 | 2.83 |  | 1.85 |  | unknown | Conserved hypothetical protein |
| AGAP003775 | 2.65 |  |  |  | unknown | Conserved hypothetical protein |
| AGAP001078 | 2.28 |  |  |  | unknown | Unknown |
| AGAP009974 | 2.12 |  |  |  | unknown | Unknown |
| AGAP008447/ AGAP008444 | 2.11 |  |  |  | unknown | Conserved hypothetical protein |
| **1DPI and 4DPI** | | |  |  |  |  |
| AGAP008118 | 0.48 | 0.35 |  |  | Cell division | Cell cycle checkpoint kinase, putative |
| AGAP004556 | 0.46 | 0.32 |  |  | Cell division | Protein peter pan, putative |
| AGAP009176 | 2.21 | 2.19 | 1.27 |  | Fatty acid synthesis | Fatty acid synthase, putative |
| AGAP003196 | 2.17 | 2.01 |  |  | Fatty acid synthesis | Elongase, putative |
| AGAP005620 | 2.02 | 3.60 | 1.66 | DPT | Immunity | Anti-microbial peptide |
| AGAP004845 | 2.01 | 3.42 |  | SCRB8 | Immunity | Scavenger receptor |
| AGAP012352 | 2.31 | 2.86 | 1.61 | ML1 | Immunity | MD2-like lipid recognition |
| AGAP009556 | 2.10 | 2.82 |  | FREP50 | Immunity | Fibrinogen-like |
| no AGAP | 2.47 | 2.77 | 1.44 | CLIPA9 | Immunity | Clip domain serine protease |
| AGAP010812 | 2.88 | 2.73 | 1.61 | TEP4 | Immunity | Thioester containing protein |
| AGAP008654 | 2.15 | 2.58 | 1.68 | TEP12 | immunity | Thioester containing protein |
| AGAP007457 | 2.10 | 2.38 |  | LRIM7 | Immunity | LRR protein |
| AGAP004455 | 2.10 | 2.55 |  | GNBPB1 | Immunity | Gram negative binding protein subgroup B |
| AGAP011790 | 2.09 | 2.36 |  | CLIPA2 | Immunity | Clip domain serine protease |
| AGAP003247 | 2.14 | 2.17 |  | CLIPB19 | Immunity | Clip domain serine protease |
| AGAP010819 | 2.53 | 2.15 |  | TEP10 | Immunity | Thioester containing protein |
| AGAP010830 | 2.23 | 2.06 |  | TEP9 | Immunity | Thioester containing protein |
| AGAP007315 | 2.04 | 2.69 |  |  | metabolism | Carbamoyl phosphate synthase protein, putative |
| AGAP005372 | 3.15 | 3.01 |  | COEBE3D | Misc | Carboxylesterase, type B |
| AGAP012320 | 2.32 | 2.48 |  | OBP25 | Misc | Odorant binding protein |
| AGAP006278 | 2.05 | 2.18 | 1.85 |  | Misc | Odorant binding protein, putative |
| AGAP008311 | 2.46 | 2.12 |  |  | Misc | Acylphophatase, putative |
| AGAP007160 | 2.12 | 2.06 |  |  | Misc | Heat shock protein,putative |
| AGAP000930 | 0.47 | 0.36 |  |  | Misc | Coronin, putative |
| AGAP006904 | 0.39 | 0.48 | 0.61 |  | Misc | Matrix metalloproteinase, putative |
| AGAP010548 | 2.21 | 2.85 | 1.61 |  | signalling | Laminin B, putative |
| AGAP001589 | 0.49 | 0.32 |  |  | Translation | Translation initiation factor eif-2b, putative |
| AGAP008306 | 2.76 | 3.58 |  |  | unknown | Conserved hypothetical protein |
| AGAP008011 | 2.10 | 3.48 |  |  | unknown | Unknown |
| AGAP006504 | 2.51 | 3.24 |  |  | unknown | Unknown |
| AGAP006507 | 2.67 | 3.00 |  |  | unknown | Unknown |
| AGAP005611 | 2.15 | 2.89 |  |  | unknown | Unknown |
| AGAP011317 | 2.05 | 2.79 |  |  | unknown | Conserved hypothetical protein |
| AGAP010066 | 2.34 | 2.70 |  |  | unknown | Conserved hypothetical protein |
| AGAP008307 | 2.44 | 2.59 |  |  | unknown | Conserved hypothetical protein |
| AGAP007711 | 2.03 | 2.44 |  |  | unknown | Conserved hypothetical protein |
| AGAP003239 | 2.42 | 2.31 | 1.26 |  | unknown | Conserved hypothetical protein |
| AGAP004208 | 0.46 | 0.43 |  |  | unknown | Conserved hypothetical protein |
| **4DPI** |  |  |  |  |  |  |
| AGAP009792 | 0.68 | 0.49 |  |  | Cell division | Importin alpha subunit, putative |
| AGAP003742 |  | 0.47 |  |  | Cell division | Regulator of chromosome condensation, putative |
| AGAP005800 |  | 0.47 |  |  | Cell division | DNA replication licensing factor MDM7 |
| AGAP006165 |  | 0.45 |  |  | Cell division | Domino, putative |
| AGAP007874 | 0.58 | 0.45 |  |  | Cell division | Initiation factor eIF-4 gamma, putative |
| AGAP007477 |  | 0.40 |  |  | Cell division | ATP dependent DNA helicase, putative |
| AGAP002440 |  | 0.34 |  | CDC42_ANOGA | Cell division | Cell division control protein |
| AGAP007112 | 0.50 | 0.27 |  |  | Cell division | Pescadillo homolog |
| AGAP010150 | 1.94 | 2.45 | 1.79 |  | Fatty acid synthesis | Cytochrome b5, putative |
| AGAP010695 |  | 2.31 |  |  | fatty acid synthesis | Elongase, putative |
| AGAP008468 | 1.88 | 2.06 | 1.57 |  | fatty acid synthesis | Fatty acid synthase, putative |
| AGAP010461 | 0.81 | 0.47 |  |  | Histone | Histone H1, putative |
| no AGAP | 1.87 | 2.86 | 1.55 | CLIPC9 | Imminuty | Clip domain serine protease |
| no AGAP | 1.65 | 2.45 |  | CLIPE4 | Immunity | Clip domain serine protease |
| AGAP008368 |  | 2.44 |  | TEP14 | Immunity | Clip domain serine protease |
| AGAP010774 |  | 2.30 |  | FREP27 | Immunity | Fibrinogen-like |
| AGAP004806 |  | 2.28 |  | GALE6 | Immunity | Galectin |
| AGAP005334 | 1.57 | 2.25 |  | CTLMA2 | Immunity | C-type lectin |
| AGAP012529 |  | 2.21 |  | GALE8 | Immunity | Galectin |
| AGAP004807 | 1.54 | 2.20 |  | GALE7 | Immunity | Galectin |
| AGAP010360 | 1.47 | 2.19 |  |  | Immunity | Peritrophin-A, putative |
| AGAP007343 |  | 2.19 |  | LYSC2 | Immunity | Lysozyme |
| AGAP005717 | 1.94 | 2.18 |  | LYSC6 | Immunity | Lysozyme |
| AGAP004920 |  | 2.14 |  | CASPS6 | Immunity | Caspase |
| AGAP003246 | 1.50 | 2.12 | 1.90 | CLIPB2 | Immunity | Clip domain serine protease |
| AGAP000694 | 1.60 | 2.11 | 1.76 | CEC3 | Immunity | Antimicrobial peptide |
| AGAP012945 | 1.58 | 2.07 |  |  | Immunity | Caspase |
| AGAP000443 | 1.73 | 2.07 |  | CTL5 | Immunity | C-type lectin |
| AGAP009166 | 0.50 | 0.48 |  | IKK1 | Immunity | I-Kappa-B Kinase-1 |
| AGAP004036 |  | 0.48 |  | HXP7 | Immunity | Peroxidase |
| AGAP007294 | 0.68 | 0.46 |  | IAP1 | Immunity | Inhibitor of apoptosis |
| AGAP004038 | 0.80 | 0.45 |  | HXP8 | Immunity | Peroxidise |
| AGAP008354 |  | 0.35 |  | HOP | Immunity | Janus kinase, Hopskotch |
| AGAP010363 |  | 4.23 |  |  | Immunity | Peritrophin-A, putative |
| AGAP010364 |  | 4.08 |  |  | Immunity | Peritrophin-A, putative |
| AGAP006707 |  | 3.23 |  |  | metabolism | Chymotrypsin, putative |
| AGAP005752 |  | 2.89 |  |  | metabolism | Glucosyl/glucuronosyl transferase, putative |
| AGAP010530 | 1.77 | 2.63 |  |  | metabolism | Chymotrypsin, putative |
| AGAP012034/AGAP012035 |  | 2.32 |  |  | metabolism | Chymotrypsin, putative |
| AGAP007142 |  | 2.29 |  |  | metabolism | Serine-type endopepsidase, putative |
| AGAP006926 |  | 2.22 |  |  | metabolism | Alcohol dehydrogenase, putative |
| AGAP012843 | 1.84 | 2.19 | 1.54 |  | metabolism | Chymotrypsin, putative |
| AGAP005124 | 1.51 | 2.18 |  |  | metabolism | Aldehyde dehydrogenase, putative |
| AGAP002721 |  | 2.16 |  |  | metabolism | Tryptophan oxygenase, putative |
| AGAP010243 |  | 2.04 |  |  | metabolism | Chymotrypsin, putative |
| AGAP007505 |  | 0.47 |  |  | metabolism | Serine carboxypeptidase, putative |
| AGAP011948 |  | 0.44 |  |  | metabolism | Threonine dehydrogenase, putative |
| AGAP002208 | 0.57 | 0.38 |  |  | Metabolism | Cytochrome P450 family, putative |
| AGAP008296 | 1.32 | 0.34 |  | TRY1_ANOGA | Metabolism | Peptidase S1A |
| AGAP007753 |  | 3.13 |  |  | Misc | Major facilitator family, sugar transporter, putative |
| AGAP007601 |  | 3.08 |  |  | Misc | Major facililtator family, general substrate transporter, putative |
| AGAP008052 |  | 2.85 |  | Q6H8Z3_ANOGA | Misc | Putative sensory appendage protein SAP-2 precursor |
| AGAP006076 | 1.44 | 2.80 |  | OBP50 | misc | Odorant binding protein |
| AGAP002198 |  | 2.73 | 1.96 |  | Misc | Methyltransferase, putative |
| AGAP010409 |  | 2.43 |  | OBP22 | Misc | Odorant binding protein |
| AGAP005837 |  | 2.37 |  |  | Misc | Juvenile hormone esterase, putative |
| AGAP007918 |  | 2.37 |  | Q8I9N3_ANOGA | Misc | Aldehyde oxidase/xanthine dehydrogenase |
| AGAP011426 |  | 2.32 |  |  | Misc | Sodium dependent phosphate transporter, putative |
| AGAP004433 | 1.67 | 2.31 |  | OBP19 | Misc | Odorant binding protein |
| AGAP002826/  AGAP002827 |  | 2.27 |  |  | Misc | Major facilitator family transporter, putative |
| AGAP008404 |  | 2.20 | 1.57 |  | Misc | Glucosyl/glucuronosyl transferase, putative |
| AGAP003733 | 1.70 | 2.18 | 1.45 |  | Misc | Phosphatidylinositol transfer protein, putative |
| AGAP008182 | 1.38 | 2.13 |  |  | Misc | Odorant binding protein, putative |
| AGAP001966 |  | 2.10 |  |  | Misc | Sodium solute symporter, putative |
| AGAP009464 | 1.36 | 2.08 | 1.53 |  | Misc | ABC transporter, putative |
| AGAP005918 | 1.70 | 2.06 |  |  | Misc | Cation efflux protein/zinc transporter, putative |
| AGAP006249 |  | 0.48 |  |  | misc | Conserved hypothetical protein, amino acid transport, putative |
| AGAP001514 |  | 0.48 |  |  | misc | Fip-1, putative |
| AGAP002622 | 0.61 | 0.48 |  |  | misc | Sodium/solute symporter, putative |
| AGAP012089 | 0.72 | 0.48 |  |  | Misc | Nuclear inhibitor of protein phosphatase-1, putative |
| AGAP004274 | 0.56 | 0.47 |  |  | misc | Conserved hypothetical, zinc finger protein, putative |
| AGAP000889 | 0.68 | 0.46 |  |  | misc | Actin-binding protein, cofilin/tropomyosin type, putative |
| AGAP005174 | 0.81 | 0.45 |  |  | Misc | Nucleoporin, putative |
| AGAP002284 |  | 0.44 |  |  | misc | N-terminal acetyltransferase, putative |
| AGAP011178 | 0.61 | 0.44 |  |  | Misc | Zinc/iron trasporter, putative |
| AGAP011250 | 0.63 | 0.43 |  |  | Misc | Igloo, putative |
| AGAP003279 | 0.60 | 0.43 |  |  | misc | Tetratrico peptide repeat protein, putative |
| AGAP009507 | 0.66 | 0.43 |  |  | Misc | Ubiquitin thioesterase, putative |
| AGAP009659 |  | 0.42 |  |  | Misc | Tetratrico peptide repeat protein, putative |
| AGAP008136 | 0.65 | 0.42 |  |  | Misc | Cyclophillin, putative |
| AGAP006925 | 0.64 | 0.42 |  |  | misc | N-acetylgalactosaminyltransferase, putative |
| AGAP006949 | 0.85 | 0.41 |  |  | misc | Metalloendopetidase, putative |
| AGAP002387 | 0.60 | 0.40 |  |  | Misc | Acid phosphatise, putative |
| AGAP011742 |  | 0.39 |  |  | Misc | N-acetyltransferase, putative |
| AGAP001423 | 0.73 | 0.38 |  |  | Misc | Bifunctional purine biosynthesis, putative |
| AGAP002409 | 0.55 | 0.36 |  |  | Misc | Pseudouridylate synthase, putative |
| AGAP001522 | 0.68 | 0.35 |  |  | Misc | Thyroid receptor interacting protein, putative |
| AGAP012005 |  | 0.27 |  |  | Misc | ABC transporter, putative |
| AGAP010303 | 1.45 | 2.23 |  |  | protein degradation | Autophagy protein,putative |
| AGAP007721 |  | 2.57 |  |  | protein transport/secretion | Synaptobrevin, putative |
| AGAP003576 |  | 0.43 |  |  | Protein transprot/secretion | Transportin, putative |
| AGAP002836 |  | 0.49 |  | Q86MA9_ANOGA (Dicer-1) | RNA degradation | Dicer-1 |
| AGAP011627 | 0.71 | 0.45 |  |  | RNA degradation | RNA metabolic process, putative |
| AGAP005672 | 0.61 | 0.34 |  | TSN | RNA degradation | Tudor-SN, putative |
| AGAP011204 | 0.57 | 0.18 |  |  | RNA degradation | PIWI, putative |
| AGAP006941 |  | 0.44 |  |  | RNA helicase | Probable ATP dependent RNA helicases |
| AGAP011145 | 0.65 | 0.42 |  |  | RNA helicase | DEAD box ATP dependent RNA helicase, putative |
| AGAP012655 |  | 0.42 |  |  | RNA helicase | Helicase, putative |
| AGAP011084 | 0.51 | 0.40 |  |  | RNA helicase | DEAD box ATP dependent RNA helicase, putative |
| AGAP009600 | 0.51 | 0.37 |  |  | RNA helicase | DEAD box ATP dependent RNA helicase, putative |
| AGAP003508 |  | 0.34 |  |  | RNA helicase | DEAD box ATP dependent RNA helicase, putative |
| AGAP011322 |  | 2.82 |  |  | Signalling | Fibulin, putative |
| AGAP010766 |  | 2.51 |  |  | signalling | Protein serine/threonine kinase, putative |
| AGAP012666 |  | 2.20 |  |  | Signalling | C-type lectin, putative |
| AGAP005719 | 0.58 | 0.48 | 0.61 |  | Signalling | Transcription initiation factor TFIID, putative |
| AGAP002035 | 0.65 | 0.47 |  |  | Signalling | DNA binding factor, putative |
| AGAP007050 | 0.69 | 0.46 |  |  | Signalling | Nucleolar GTP binding protein,putative |
| AGAP005289 |  | 0.45 |  |  | Signalling | Myostatin, putative |
| AGAP003121 | 0.68 | 0.45 |  |  | Signalling | PI-4 kinase, putative |
| AGAP001953 | 0.71 | 0.42 |  |  | Signalling | Rab GTPase activator,putative |
| AGAP000627 | 0.67 | 0.42 |  |  | Signalling | Casein kinase 1 isoform, putative |
| AGAP007118 |  | 0.42 |  |  | Signalling | Tyrosine phosphatase,putative |
| AGAP005362 | 0.64 | 0.39 |  |  | Signalling | X-box binding protein, putative |
| AGAP002902 | 0.54 | 0.36 |  | Q6PUC1_ANOGA (medea) | Signalling | Smad-4 |
| AGAP002123 | 0.55 | 0.36 |  |  | Signalling | Axin, putative |
| AGAP008149 | 0.58 | 0.35 |  |  | Signalling | Protein phosphatise, putative |
| AGAP010984 | 0.65 | 0.33 |  |  | Signalling | Transcription factor TFIIH, putative |
| AGAP010118 |  | 2.32 |  |  | Transcription | Histone acetyltransferase, putative |
| AGAP001900 |  | 0.49 |  |  | Transcription | Abo, putative |
| AGAP009024 | 0.63 | 0.28 |  |  | Transcription | RNA polymerase I specific transcription initiation factor RRN3, putative |
| AGAP001879 | 0.66 | 0.49 |  |  | Translation | Crooked neck protein, putative |
| AGAP005640 | 0.60 | 0.49 |  |  | Translation | Pre mRNA spicing factor, putative |
| AGAP007326 | 0.76 | 0.47 |  |  | Translation | Lsm11, putative |
| AGAP000325 | 0.84 | 0.46 |  |  | Translation | Lysyl tRNA synthetase, putative |
| AGAP002337 | 0.68 | 0.43 |  |  | Translation | Eukaryotic translation initiation factor 3, subunit 7 |
| AGAP004336 | 0.51 | 0.40 |  |  | Translation | U3 small nucleolar RNA-associated protein, putative |
| AGAP012283 | 0.64 | 0.34 |  |  | Translation | Cysteinyl-tRNA synthetase, putative |
| AGAP006200 |  | 4.28 |  |  | unknown | Conserved hypothetical protein |
| AGAP003083 | 1.31 | 2.98 |  |  | unknown | Conserved hypothetical protein |
| AGAP006506 | 1.97 | 2.82 |  |  | unknown | Unknown |
| AGAP010365 |  | 2.82 |  |  | unknown | Conserved hypothetical protein |
| AGAP004784 | 1.69 | 2.80 |  |  | unknown | Conserved hypothetical protein |
| AGAP007946 |  | 2.64 |  |  | unknown | Conserved hypothetical protein |
| AGAP002582 | 1.35 | 2.60 |  |  | unknown | Conserved hypothetical protein |
| AGAP010111 |  | 2.52 |  |  | unknown | Unknown |
| AGAP002853 |  | 2.46 |  |  | unknown | Unknown |
| AGAP005614 | 1.90 | 2.34 |  |  | unknown | Conserved hypothetical protein |
| AGAP012432 | 1.37 | 2.30 |  |  | unknown | Conserved hypothetical protein |
| AGAP004549 |  | 2.29 |  |  | unknown | Conserved hypothetical protein |
| AGAP010502 |  | 2.29 |  |  | unknown | Unknown |
| AGAP010385 |  | 2.27 |  |  | unknown | Unknown |
| AGAP003095 | 1.92 | 2.26 |  | Q8MZM5_ANOGA | unknown | Conserved hypothetical protein |
| AGAP008013 | 1.67 | 2.25 |  |  | unknown | Conserved hypothetical protein |
| AGAP005247 |  | 2.24 |  |  | unknown | Unknown |
| AGAP004695 | 1.48 | 2.24 |  |  | unknown | Conserved hypothetical protein |
| AGAP006259 | 1.77 | 2.23 |  |  | unknown | Unknown |
| AGAP011784 | 1.80 | 2.22 |  |  | unknown | Unknown |
| AGAP008301 |  | 2.22 |  |  | unknown | Unknown |
| AGAP004674 | 1.51 | 2.13 |  |  | unknown | Conserved hypothetical protein |
| AGAP003636 |  | 2.11 |  |  | unknown | Conserved hypothetical protein |
| AGAP009145 | 1.48 | 2.09 |  |  | unknown | Unknown |
| AGAP001989 |  | 2.07 |  |  | unknown | Unknown |
| AGAP012436 |  | 2.05 |  |  | unknown | Unknown |
| AGAP006275 | 1.54 | 2.04 |  |  | unknown | Conserved hypothetical protein |
| AGAP005259 | 0.62 | 0.48 |  |  | unknown | Conserved hypothetical protein |
| AGAP011333 |  | 0.48 |  |  | unknown | Conserved hypothetical protein |
| AGAP003086 | 0.73 | 0.47 |  |  | unknown | Conserved hypothetical protein |
| AGAP002014 | 0.65 | 0.46 |  |  | unknown | Conserved hypothetical protein |
| AGAP003646 | 0.62 | 0.46 |  |  | unknown | Conserved hypothetical protein |
| AGAP005033 | 0.62 | 0.46 |  |  | unknown | Conserved hypothetical protein |
| AGAP004468 | 0.65 | 0.45 |  |  | unknown | Conserved hypothetical protein |
| AGAP012103 | 0.57 | 0.45 |  |  | unknown | Conserved hypothetical protein |
| AGAP009424 |  | 0.44 |  |  | unknown | Conserved hypothetical protein |
| AGAP012281 |  | 0.42 |  |  | unknown | Conserved hypothetical protein |
| AGAP009307 |  | 0.40 |  |  | unknown | Conserved hypothetical protein |
| AGAP008879 |  | 0.39 |  |  | unknown | Conserved hypothetical protein |
| AGAP004463 | 0.56 | 0.34 |  |  | unknown | Conserved hypothetical protein |
| AGAP000270 | 0.63 | 0.32 |  |  | Unknown | Conserved hypothetical protein |
| **4DPI and 9DPI** | | |  |  |  |  |
| AGAP005693 |  | 5.88 | 2.76 |  | Immunity | LRR protein |
| AGAP001768 |  | 3.16 | 2.35 |  | metabolism | GILT-like protein |
| AGAP008141 |  | 3.25 | 2.03 |  | Misc | Fumerate lyase, putative |
| AGAP003776 |  | 8.33 | 2.81 |  | unknown | Conserved hypothetical protein |
| **9DPI** | | |  |  |  |  |
| AGAP003502 | 1.45 |  | 2.53 | HPX6 | Immunity | Peroxidase |
| AGAP012037 |  |  | 2.26 | CLIPB20 | Immunity | Clip domain serine protease |
| AGAP003246 |  |  | 2.18 | CLIPB2 | Immunity | Clip domain serine protease |
| AGAP004316 | 1.91 |  | 2.72 |  | immunity? | CLIP |
| AGAP004880 |  |  | 3.43 |  | metabolism | L-lactate dehydrogenase, putative |
| AGAP012561 | 1.62 |  | 2.98 |  | misc | Peroxidise, putative |
| AGAP009551 |  |  | 2.26 |  | misc | Sulfotransferase, putative |
| AGAP005458 |  |  | 0.43 |  | misc | 5'nucleotidase, putative |
| AGAP008862 |  |  | 0.50 |  | RNA degradation | Ago3, putative |
| AGAP007965 |  |  | 0.49 |  | RNA degradation | maternal tudor domain containing, putative |
| AGAP005079 | 1.43 | 1.66 | 2.20 | AOEJE7_ANOGA | Signalling | G protein alpha subunit q2 |
| AGAP001610 |  | 1.83 | 2.44 |  | unknown | Conserved hypothetical protein |
| AGAP004133 |  |  | 2.41 |  | unknown | Conserved hypothetical protein |
| AGAP012604 |  |  | 2.41 |  | unknown | Conserved hypothetical protein |
| AGAP001096 | 1.21 | 1.59 | 2.38 |  | unknown | Conserved hypothetical protein |
| **1DPI and 9DPI** | | |  |  |  |  |
| AGAP005848 | 2.05 | 1.85 | 2.59 | FREP44 | Immunity | Fibrinogen-like |
| AGAP010781 | 2.64 |  | 2.46 |  | unknown | Conserved hypothetical protein |
| AGAP000183 | 2.45 |  | 2.07 |  | unknown | Unknown |
| **1DPI, 4DPI and 9DPI** | | |  |  |  |  |
| AGAP007385 | 2.03 | 2.54 | 2.07 | LYSC4 | Immunity | Lysozyme |

**Genes differentially regulated by 5’ONNVic-eGFP infection** . Two-fold or greater fold change ratios are shown in black text for 1DPI, 4DPI and 9DPI. Fold change ratios less than 2-fold regulated that have passed all filters outlines in the materials and methods excluding filtering on fold change ratio, are shown in grey text. Putative functions/functional domains were derived from Gene ontology terms, Interpro domains and functions of orthologous genes ([www.vectorbase.org](http://www.vectorbase.org/)).
