# Supplementary material for: Anopheles gambiae Antiviral Immune Response to Systemic O'nyong-nyong Infection
Source: PLoS Negl Trop Dis. 2012 Mar 13;6(3):e1565. doi: 10.1371/journal.pntd.0001565 (PMC3302841; doi:10.1371/journal.pntd.0001565)
Supplement: Table S2 — Immune genes regulated by viral infection at days 1,4 and 9 post infection. Two-fold or greater fold change ratios are shown in black text for 1DPI, 4DPI and 9DPI).. Fold change ratios less than 2-fold regulated that have passed all filters outlines in the materials and methods excluding filtering on fold change ratio, are shown in grey text. Putative functions/functional domains were derived from Gene ontology terms, Interpro domains and functions of orthologous genes (www.vectorbase.org). (DOCX) [file pntd.0001565.s003.docx]

**Table S2. Immune genes regulated by viral infection at days 1,4 and 9 post infection.**

|  |  |  | **Fold change ratio** | | |  |
| --- | --- | --- | --- | --- | --- | --- |
| **Gene** | **Functional group** | **Name** | **Day 1** | **Day 4** | **Day 9** | **putative function** |
| **1DPI** | | | | | | |
| AGAP010814 | Complement | TEP5 | 2.60 |  |  | Thioester containing protein, complement |
| AGAP006348 | Complement | LRIM1 | 2.28 |  |  | LRIM protein, complement [[23](#_ENREF_23)] |
| AGAP004248 | Effector | GPXH3 | 2.27 |  |  | peroxidase, GPX sub familiy |
| AGAP002848 | Recognition | ML9 | 2.01 |  |  | MD2-like lipid recognition |
| AGAP007039 | Complement | LRIM4 | 2.68 |  |  | LRIM protein, complement |
| **1DPI and 4DPI** | | | | | | |
| AGAP010812 | Complement | TEP4 | 2.89 | 2.48 | 1.61 | Thioester containing protein, complement [[41](#_ENREF_41)] |
| AGAP008654 | Complement | TEP12 | 2.15 | 2.36 |  | Thioester containing protein, complement |
| AGAP010819 | Complement | TEP10 | 3 | 2.10 |  | Thioester containing protein, complement |
| AGAP010830 | Complement | TEP9 | 2 | 2.06 |  | Thioester containing protein, complement |
| AGAP005620 | Effector | DPT | 2.02 | 2.92 |  | Anti-microbial peptide |
| AGAP011790 | Melanisation | CLIPA2 | 2.09 | 2.08 |  | Clip domain serine protease, inhibitor of melanisation [[39](#_ENREF_39)] |
| AGAP012352 | Recognition | ML1 | 2.31 | 2.70 |  | MD2-like lipid recognition [[41](#_ENREF_41)] |
| AGAP009556 | Recognition | FREP50 | 2.15 | 2.71 |  | Fibrinogen like, function unknown |
| AGAP007457 | Complement | LRIM7 | 2.1 | 2.31 |  | LRIM protein, complement |
| AGAP004455 | Recognition | GNBPB1 | 2.10 | 2.45 |  | Gram negative binding protein, subgroup B |
| AGAP004845 | Opsonisation | SRCB8 | 2.01 | 3.13 |  | Scavenger receptor, cell adhesion |
| AGAP010968 | Signal modulation | CLIPA9 | 2.47 | 2.64 |  | Clip domain serine protease, function unknown |
| AGAP003247 | Signal modulation | CLIPB19 | 2.14 | 2.17 |  | Clip domain serine protease, function unknown |
| **1DPI and 9DPI** | | | | | | |
| AGAP005848 | Recognition | FREP44 | 2.05 |  | 2.59 | Fibrinogen-like, function unknown |
| **4DPI** | | | | | | |
| AGAP004920 | Apoptosis | CASPS6 |  | 2.05 |  | Caspase, promoter of apoptosis |
| AGAP007294 | Apoptosis | IAP1 |  | 0.48 |  | Inhibitor of apoptosis |
| AGAP008368 | Complement | TEP14 |  | 2.35 |  | Thioester containing protein, complement |
| AGAP005717 | Effector | LYSC6 |  | 2.10 |  | Lysozyme, lysis of pathogens |
| AGAP007343 | Effector | LYSC2 |  | 2.08 |  | Lysozymes, lysis of pathogens |
| AGAP000694 | Effector | CEC3 |  | 2.04 |  | Anti-microbial peptide |
| AGAP004036 | Effector | HPX7 |  | 0.49 |  | Peroxidase |
| AGAP004038 | Effector | HPX8 |  | 0.48 |  | Peroxidase |
| AGAP009166 | IMD pathway | IKK1 |  | 0.49 |  | IMD pathway component |
| AGAP008354 | JAK/STAT pathway | HOP |  | 0.41 |  | Janus kinase of JAK/STAT pathway |
| AGAP005334 | Melanisation | CTLMA2 |  | 2.10 |  | C-type lectin, inhibitor of melanisation |
| AGAP004846 | Opsonisation | SRCB9 |  | 2.21 |  | Scavenger receptor, cell adhesion, |
| AGAP007455 | Complement | LRIM10 |  | 2.96 |  | LRIM protein, function unknown |
| AGAP004806 | Recognition | GALE6 |  | 2.20 |  | Galectin, sugar binding |
| AGAP004807 | Recognition | GALE7 |  | 2.16 |  | Galectin, sugar binding |
| AGAP010774 | Recognition | FREP27 |  | 2.13 |  | Fibrinogen-like, function unknown |
| AGAP012529 | Recognition | GALE8 |  | 2.08 |  | Galectin, sugar binding |
| N/A | Signal modulation | CLIPC9 |  | 2.59 |  | Clip domain serine protease, function unknown |
| N/A | Signal modulation | CLIPE4 |  | 2.33 |  | Clip domain serine protease, function unknown |
| AGAP000443 | Signal modulation | CTL5 |  | 2.04 |  | C-type lectin, function unknown |
| AGAP005672 | RNAi | TSN | 0.61 | 0.34 |  | RNA degradation, classical RNAi pathway |
| **4DPI and 9DPI** | | | | | | |
|  |  |  |  |  |  |  |
| AGAP005693 | Complement | LRIM17 |  | 3.90 | 2.76 | LRIM protein, complement |
| AGAP003246 | Signal modulation | CLIPB2 |  | 2.05 | 2.18 | Clip domain serine protease, function unknown |
| **9DPI** | | | | | | |
| AGAP012037 | Signal modulation | CLIPB20 |  |  | 2.26 | Clip domain serine protease, function unknown |
| AGAP003502 | Effector | HPX6 |  |  | 2.55 | Peroxidase |
| **1DPI, 4DPI and 9DPI** | | | | | | |
| AGAP007385 | Effector | LYSC4 | 2.03 | 2.37 | 2.04 | Lysozyme, lysis of pathogens |

**Immune genes regulated by viral infection at days 1,4 and 9 post infection.** Two-fold or greater fold change ratios are shown in black text for 1DPI, 4DPI and 9DPI).. Fold change ratios less than 2-fold regulated that have passed all filters outlines in the materials and methods excluding filtering on fold change ratio, are shown in grey text. Putative functions/functional domains were derived from Gene ontology terms, Interpro domains and functions of orthologous genes ([www.vectorbase.org](http://www.vectorbase.org))
