# Supplementary material for: Anopheles gambiae Antiviral Immune Response to Systemic O'nyong-nyong Infection
Source: PLoS Negl Trop Dis. 2012 Mar 13;6(3):e1565. doi: 10.1371/journal.pntd.0001565 (PMC3302841; doi:10.1371/journal.pntd.0001565)
Supplement: Table S3 — Primers used for A. qrt-PCR and B. RNAi probe generation. A. Primers were designed using Primer3 for 50–150 bp sections of genes of interest. B. Primers were designed for 200–600 bp sections of genes of interest, with a T7 promotor sequence (GAATTAATACGACTCACTATAGGGAGA) added to their 5′ ends, ensuring no overlap between sections used for designing qrt-PCR primers. (DOC) [file pntd.0001565.s004.doc]

Table S3. Primers used for A. qrt-PCR and B. RNAi probe generation.

| Gene | Gene Name | F primer | R primer |
| --- | --- | --- | --- |
| **A** |  |  |  |
| n/a | nsP3 | TGA TGA AGC AGT GCC AGT TC | CGA ATT TGC GTA CAT TGG TG |
| AGAP012352 | ML1 | GTCGCTATTGTGGCATTGTG | AAAGTTTACTACTTCTGCCCAAGC |
| AGAP012529 | GALE8 | CTGCATACAGTCCGCCAAC | GTCCGCGAATCGTAATCTTG |
| AGAP007385 | LYSC4 | GATATCGAGTGTGCGAAGCA | CAGATCGGGCAGTGTCTTTT |
| AGAP010592 | S7 | GTGCGCGAGTTGGAGAAGA | ATCGGTTTGGGCAGAATGC |
| **B** |  |  |  |
| n/a | LacZ | GAATTAATACGACTCACTATAGGGAGAATCCGACGGGTTGTTACT | GAATTAATACGACTCACTATAGGG CACCACGCTCATCGATAATTT |
| AGAP008354 | HOP | GAATTAATACGACTCACTATAGGGAGAGCCAAGGAGCTGGTTATCAA | GAATTAATACGACTCACTATAGGGAGAAGAGCAGATCGTGCTTGGTT |
| AGAP011537 | Ago2 | GAATTAATACGACTCACTATAGGGAGAGCATGAGCACGCTCAACAAC | GAATTAATACGACTCACTATAGGGAGAGTTCGAGTCGTCGTACAGCA |
| n/a | nsP3 | GAATTAATACGACTCACTATAGGGAGAACCGGTGTGTACTCAGGAGG | GAATTAATACGACTCACTATAGGGAGACATAGGCACGGGACTGTTTT |
| AGAP002836 | Dicer1 | GAATTAATACGACTCACTATAGGGAGATGCTAAGCTTTGGCTGGAAT | GAATTAATACGACTCACTATAGGGAGATTTCGTTCGACCATGTACCA |
| AGAP006941 | STAT1 | GAATTAATACGACTCACTATAGGGAGAGAAAATCAACCACCGCAAGT | GAATTAATACGACTCACTATAGGGAGACAGCTCTTCCTGTTCCAAGG |
| AGAP003508 | STAT2 | GAATTAATACGACTCACTATAGGGAGACATGAACAACATCGGCAATC | GAATTAATACGACTCACTATAGGGAGATGCATATTCTCTGCCGTGAG |
| AGAP005031 | PIAS | GAATTAATACGACTCACTATAGGGAGAAATCCAATTCCCACCAACAA | GAATTAATACGACTCACTATAGGGAGAGGACAGTTCCATGTTGGCTT |
| AGAP009515 | REL1 | GAATTAATACGACTCACTATTAGGGAGAATCAACAGCACGACGATGAG | GAATTAATACGACTCACTATTAGGGAGATCGAAAAAGCGCACCTTAATT |
| AGAP007938 | CACTUS | GAATTAATACGACTCACTATTAGGGAGAGTCCGCTCTACACATCAGCA | GAATTAATACGACTCACTATTAGGGAGACCGTTCGGGTTAATGATGAC |
| AGAP006747 | REL2 | GAATTAATACGACTCACTATTAGGGAGAAATCCGACGCAAAGATACG | GAATTAATACGACTCACTATTAGGGAGAGACCGCAATGTGAAGGATG |
| AGAP012352 | ML1 | GAATTAATACGACTCACTATAGGGAGAGAAATGTCCCGGTGAAGAGA | GAATTAATACGACTCACTATAGGGAGACCCACCAGCGTTGTTTTAGT |
| AGAP002848 | ML9 | GAATTAATACGACTCACTATAGGGAGAACGGTCCATAGCAAGGATTG | GAATTAATACGACTCACTATAGGGAGAGTCAGCGGACAGGAAGTGTT |
| AGAP012529 | GALE8 | GAATTAATACGACTCACTATAGGGAGAGGTCTAGGCATTTACCGCAA | GAATTAATACGACTCACTATAGGGAGAGAGCCGTCCTTATTCTGTGG |
| AGAP007343 | LYSC2 | GAATTAATACGACTCACTATAGGGAGAAAGAAATTGTTGCCGGATTG | GAATTAATACGACTCACTATAGGGAGAGATGACGACAGGCTACAGCA |
| AGAP007385 | LYSC4 | GAATTAATACGACTCACTATAGGGAGAGAAGACGGTGAATCGGGTAA | GAATTAATACGACTCACTATAGGGAGAGTCGTTCAGAAAGTCCTCGC |
| AGAP005717 | LYSC6 | GAATTAATACGACTCACTATAGGGAGATGACATCTACTGGTGCTCGC | GAATTAATACGACTCACTATAGGGAGAAACTCACTCCACAAGCCCAC |
| AGAP000694 | CEC3 | GAATTAATACGACTCACTATAGGGAGAGAGATCTCTTCCCGTGTGGA | GAATTAATACGACTCACTATAGGGAGAGCGGTGACCTCTTTCAGTCT |
| AGAP008654 | TEP12 | GAATTAATACGACTCACTATAGGGAGAACAAGCTCTAACCTTCGCCA | GAATTAATACGACTCACTATAGGGAGAAGCACTTTGTTGCCTTGCTT |
| AGAP006941 | Helicase 2 | GAATTAATACGACTCACTATAGGGAGATCTCATCCCACGATCATTCA | GAATTAATACGACTCACTATAGGGAGAACCACTATGTCGACCTTCGG |
| AGAP003508 | Helicase 3 | GAATTAATACGACTCACTATAGGGAGAAAGGGGGAGAAAGAGATGGA | GAATTAATACGACTCACTATAGGGAGAGGCGCACAGCAGAATATGTA |

**Primers used for** A**. qrt-PCR and** B**. RNAi probe generation. A.** Primers were designed using Primer3 for 50-150bp sections of genes of interest. **B.** Primers were designed for 200-600bp sections of genes of interest, with a T7 promotor sequence (GAATTAATACGACTCACTATAGGGAGA) added to their 5′ ends, ensuring no overlap between sections used for designing qrt-PCR primers.
